# Survival of *Staphylococcus aureus* on the outer shell of fire fighter turnout gear after sanitation in a commercial washer/extractor

**DOI:** 10.1186/s12995-019-0230-4

**Published:** 2019-03-22

**Authors:** Daniel Farcas, Francoise M. Blachere, Michael L. Kashon, Deborah Sbarra, Diane Schwegler-Berry, Jeffrey O. Stull, John D. Noti

**Affiliations:** 1 Allergy and Clinical Immunology Branch, Health Effects Laboratory Division, National Institute for Occupational Safety and Health, Centers for Disease Control and Prevention, 1095 Willowdale Road M/S L-4020, Morgantown, West Virginia 26505-2888 USA; 2 Department of Occupational and Environmental Health Sciences, West Virginia University, Morgantown, West Virginia 26505 USA; 3 Biostatistics and Epidemiology Branch, National Institute for Occupational Safety and Health, Centers for Disease Control and Prevention, 1095 Willowdale Road, Morgantown, West Virginia 26505 USA; 4 National Personal Protective Technology Laboratory, National Institute for Occupational Safety and Health, Centers for Disease Control and Prevention, 1095 Willowdale, Road, Morgantown, West Virginia 26505 USA; 5 Pathology and Physiology Research Branch, Health Effects Laboratory Division, National Institute for Occupational Safety and Health, Centers for Disease Control and Prevention, 1095 Willowdale Road, Morgantown, West Virginia 26505 USA; 6 International Personnel Protection, Inc., Box 92493, Austin, TX 78709 USA

**Keywords:** Fire fighting, MRSA, MSSA, Contact transmission, Sanitizer, Biofilm

## Abstract

**Background:**

Methicillin-resistant *Staphylococcus aureus* contamination on surfaces including turnout gear had been found throughout a number of fire stations. As such, the outer shell barrier of turnout gear jackets may be an indirect transmission source and proper disinfection is essential to reduce the risk of exposure to fire fighters. Cleaning practices vary considerably among fire stations, and a method to assess disinfection of gear washed in commercial washer/extractors is needed.

**Methods:**

Swatches (1 in. ×  1.5 in.) of the outer shell fabrics, Gemini™, Advance™, and Pioneer™, of turnout gear were inoculated with *S. aureus*, and washed with an Environmental Protection Agency-registered sanitizer commonly used to wash turnout gear. To initially assess the sanitizer, inoculated swatches were washed in small tubes according to the American Society for Testing Materials E2274 Protocol for evaluating laundry sanitizers. Inoculated swatches were also pinned to turnout gear jackets and washed in a Milnor commercial washer/extractor. Viable *S. aureus* that remained attached to fabric swatches after washing were recovered and quantified. Scanning Electron Microscopy was used to characterize the stages of *S. aureus* biofilm formation on the swatches that can result in resistance to disinfection.

**Results:**

Disinfection in small tubes for only 10 s reduced the viability of *S. aureus* on Gemini™, Advance™, and Pioneer™ by 73, 99, and 100%, respectively. In contrast, disinfection of *S. aureus*-contaminated Gemini™ swatches pinned to turnout gear and washed in the washer/extractor was 99.7% effective. Scanning Electron Microscopy showed that biofilm formation begins as early as 5 h after attachment of *S. aureus*.

**Conclusion:**

This sanitizer and, likely, others containing the anti-microbial agent didecyl dimethyl ammonium chloride, is an effective disinfectant of *S. aureus.* Inclusion of contaminated outer shell swatches in the wash cycle affords a simple and quantitative method to assess sanitization of gear by commercial gear cleaning facilities. This methodology can be extended to assess for other bacterial contaminants. Sanitizer-resistant strains will continue to pose problems, and biofilm formation may affect the cleanliness of the washed turnout gear. Our methodology for assessing effectiveness of disinfection may help reduce the occupational exposure to fire fighters from bacterial contaminants.

## Background

*Staphylococcus aureus* is an opportunistic bacterial pathogen that can cause a diversity of medical complications ranging from skin infections to sepsis, pneumonia and death [1]. Timely treatment of both methicillin sensitive *S. aureus* (MSSA) and methicillin resistant *S. aureus* with appropriate antibiotics typically resolves infection. However, MRSA bloodstream infections have higher mortality compared to MSSA bloodstream infections [2]. The Centers for Disease Control and Prevention (CDC) estimates that MRSA has caused over 80,000 infections and 11,285 deaths in the United States in 2011 [3]. Infection by MRSA is further classified into hospital-associated MRSA (HA-MRSA) and community-acquired MRSA (CA-MRSA). The high rate of EMS responses by fire fighters increases the likelihood that they will be exposed to both variants of MRSA as they work in both hospital and communal environments. In particular, the communal lifestyles of fire fighters poses a particular risk [4]. In that report, cases of MRSA were confirmed in the Phoenix, AZ (29 cases 2004–2006), Los Angeles, CA (50 cases 2003–2006), and Mesa, AZ (two case in 2001 and 2006) fire stations. The detection of MRSA on surfaces throughout the training and communal areas of nine Arizona fire facilities suggested fomite transmission of this pathogen [5]. A subsequent study collected 1064 environmental samples from two fire stations in two northwest United States fire districts [6]. In that study, of the 44 samples (4.1%) that were MRSA-positive, five samples were from outer fire gear. Further, nine MRSA-positive nasal samples (from four fire stations of one district) were obtained from fire station personnel and some were genetically related to the environmental samples and suggests possible fomite transmission within the stations. In a third study, environmental surface sampling in 33 Washington state fire stations revealed that 27 stations (82%) were MSSA positive and 14 stations (58%) were positive for both MRSA and MSSA [7]. In that study, of the 119 MSSA positive and 52 MRSA positive environmental samples, seven (5.9%) outer shells of fire fighter turnout gear were MSSA positive and five (9.6%) were MRSA positive. These studies suggest that soiled and/or improperly cleaned turnout gear may indirectly transmit *S. aureus*. It has been demonstrated that staphylococci bacteria can survive for months (> 90 days) after drying on hospital linens [8] and even up to 7 months on surfaces [9], increasing the likelihood of indirect transmission in communal living areas, and placing fire fighters at an elevated risk for *S. aureus* infection.

The Mesa Fire Department suggested that keeping firefighting turnout gear clean is essential to limiting MRSA infections and instituted an in-house cleaning program with dedicated washers/dryers for turnouts [4]. The Colorado Department of Public Health urged the Boulder Fire Department to prohibit sharing of turnouts and helmets by fire fighters to prevent the spread of MRSA and suggested that each fire fighter have a second set of clean gear available during wash cycles [10]. In accordance with the National Fire Protection Association (NFPA) 1851: Standard on Selection, Care, and Maintenance of Protective Ensembles for Structural Firefighting and Proximity Firefighting, it is recommended that firefighting turnout gear be laundered at least twice per year [11]. Moreover, the NFPA 1851 provides guidelines on cleaning turnout gear with antimicrobials while maintaining fabric integrity. However, there is no verification that the established procedures, often modified by industry care providers, adequately remove contaminants from fire fighter gear. Manufacturers of cleaning procedures and cleaning claims by independent service providers (ISPs) that wash turnout gear have not been substantiated. To address this need, a task group was formed under the Technical Committee on Structural and Proximity Fire Fighting Protective Clothing and Equipment for the revision of NFPA 1851 to develop cleaning and validation procedures for the fire fighting industry.

The outer shells of turnout gear are water and chemical-resistant and used as the initial barrier to prevent contact of the fire fighter’s body with harmful reagents. The most commonly used outer shell fabric is Gemini™ followed by Advance™ and of late, a new fabric, Pioneer™, has been introduced for serious consideration. Various sanitizers are in use by ISPs to disinfect these materials, however, their efficiencies have not been evaluated. An EPA-registered sanitizer containing the anti-microbial agent, didecyl dimethyl ammonium chloride (DDAC), and that is commonly used by ISPs was chosen for analysis in this study. Using the methods outlined in the ASTM E2274 [12], ASTME1054 [13], and NFPA 1851 [11], the purpose of this study was to [1] evaluate the efficacy of this sanitizer to disinfect *S. aureus* contamination on these outer shell fabrics, and [2] develop a simple yet quantitative methodology that can be used by ISPs that launder fire fighter turnout gear to verify *S. aureus* decontamination. The methodology described can easily be adapted to test the ISP’s wash protocol to decontaminate a wide variety of microbial species.

## Methods

### Bacterial strain and growth conditions

*S. aureus* subsp. *aureus* Rosenbach (ATCC® 6538™) was purchased from the American Type Culture Collection (ATCC, Manassas, VA) and cultured in tryptic soy broth (TSB) as specified by the ATCC. *S. aureus* growth curves were established according to Benson’s Microbiological Applications [14]. TSB and tryptic soy agar (TSA) (Sigma-Aldrich, St. Louis, MO) were also used to assess bacterial contamination prior to sterilization of outer shell fabric. A DDAC resistant strain of *Pseudomonas aeruginosa* was recovered as a contaminant on a *S. aureus* inoculated swatch of outer shell fabric following a test wash of this swatch along with decommissisoned turnout jackets in an in-house commercial washer/extractor. The identity of the DDAC-resistant *P. aeruginosa* strain was determined by its colony morphology on nutrient agar (NA), pellicle formation on the surface of Dey-Engley Broth (DEB) (Sigma-Aldrich), and under the scanning electron microscopy (SEM), and by Quantitative Real-Time Polymerase Chain Reaction (qPCR) detection of the *regA* gene.

### Investigated fabrics and turnout gear

Firefighting turnout gear jackets and pants are fabricated as a three-layer composite system. The brands of outer layer examined were: fully-finished TenCate (TenCate Protective Fabrics, Union City, GA) Gemini™ XT (40% polybenzimidazole and 60% DuPont™ Kevlar® blend spun yarn with Super Shelltite water repellent finish and nominal weight 7.5 oz./yd^2^), fully finished TenCate Advance™ (40% DuPont™ Nomex® and 60% DuPont™ Kevlar® blend spun yarn with Super Shelltite water repellent finish and nominal weight 7.0 oz./yd^2^) and scoured (no dyes or water repellant finish) TenCate Pioneer™ with ENFORCE™ Technology (DuPont™ Nomex® and Kevlar® blend spun yarn and nominal weight 6.6 oz./yd^2^) which was not dyed or treated with finishing chemicals by the manufacturer. Gemini™ was laundered in-house five times prior to its use in experiments, Advance™ was received already laundered (10 cycles according to an industry standard specified in the NFPA 1851 turnout clothing standard), and Pioneer™ was not laundered before use. Prior to experimentation, each fabric was tested for bacterial contamination by incubating fabric swatches at 37 °C in TSB for 5 h followed by dilution plating on TSA plates. For washer/extractor tests, decommissioned turnout gear (three jackets and three pants) were used. All fabrics and turnout gear used in this study were kindly provided by Tim Tomlinson from Gear Cleaning Solutions, LLC Dallas, TX. The examined fabric standard was the American Association of Textile Colourists and Chemists (AATCC) ballast 100% cotton cat. no: KCT-3011.

### Fabric sterilization and scanning electron microscopy (SEM)

Outer shell fabrics provided by the manufacturer were examined under SEM for microbial contamination and structural changes before and after scouring (as per ASTM E2274 procedure) or autoclaving (30 min at 121 °C). For SEM, 0.8 cm^2^ pieces of fabric were placed into a 24 well cell culture plate, fixed in formalin/osmium tetroxide, dehydrated with ethanol, dried using hexamethyldisalizane, mounted onto aluminum stubs and sputter-coated with gold palladium. The samples were imaged on a Hitachi S-4800 Field Emission Scanning Electron Microscope operated at 5 kV. Each sample was first scanned at 500X for the presence of bacterial cells and photographs were taken at 20,000X magnification.

### Bacteriocidal efficacy of the sanitizer

The sanitizer used contains 50% DDAC, an antimicrobial quaternary ammonium chemical. To assess the bactericidal efficacy of the sanitizer, sterile culture tubes (ThermoFisher Scientific) containing 2 ml of sanitizer diluted 1:6803 in water were inoculated with 1.9 × 10^7^viable *S. aureus* that were in logarithmic stage at 0.6 OD_600._ Tubes containing 2 ml sterile Milli-Q water and 1.9 × 10^7^ *S. aureus* served as control. The tubes were vortexed for either 10 s, 1 min or 10 min, and 2 ml of DEB was immediately added to neutralize the sanitizer [15]. Neutralized cell suspensions were dilution plated in triplicate on TSA. Optimization of DEB neutralization was previously determined according to the protocol in ASTM E1054. Briefly, 100 *S. aureus* colony forming units (CFUs) were diluted into DEB and either sanitizer or phosphate buffered saline (PBS) was then added to the mixture. The suspended cells were either immediately plated onto TSA plates, or allowed to stand for 1 or 10 min before plating. As controls, cells were resuspended in PBS or sanitizer, and either immediately plated or allowed to stand for 1 or 10 min before plating. The addition of 2 ml DEB completely inactivated the sanitizer at all time points. Addition of DEB did not reduce viability of cells when compared to cells plated without DEB exposure.

### Quantitative polymerase chain reaction (qPCR) analysis

Bacterial quantification was performed using the Primerdesign™ genesig® Advanced Kits for *S. aureus* (targets the *femB* chromosomal gene) or *P. aeruginosa* (targets the *regA* chromosomal gene) (Primerdesign LTD, Cambridge, United Kingdom). All qPCR reactions and determinations of the limit of detection (LOD) and limit of quantitation (LOQ) were performed using the 7500 Fast Real Time PCR System (Applied Biosystems, ThermoFisher Scientific, Pittsburg, PA) according to Blachere et al. [16]. Genomic DNA extraction was performed using DNeasy® Blood and Tissue Kit (QIAGEN, Valencia, California) including the manufacturer’s optional heat step at 95–100 °C, 15 min. The final eluted DNA volume was 50 μl and 5 μl DNA was used in the qPCR (*S. aureus*: LOD = 9 gene copies, LOQ =11 gene copies. *P. aeruginosa*: LOD =5 gene copies, LOQ =10 gene copies).

### Effectiveness of fabric sanitization using ASTM E2274 protocol

The ASTM E3374 Protocol was initially used to simulate machine washing of laundered outer shell fabrics. Three Gemini™ or Advance™ swatches (1 in. ×  1.5 in./25.4 mm × 38.1 mm) and laundered as above) inoculated with *S. aureus* were aseptically sandwiched inside a Gemini™ fabric wrapped spindle, and the spindle was placed in a 1000 ml canister containing 150 ml of diluted sanitizer or water, and rotated at 60 RPM/min for 10 min at room temperature (~ 20 °C). The protocol for wrapping the spindle and insertion of swatches inside the Gemini™ fabric is as per ASTM2274 [12]. Sandwiching swatches between layers of Gemini™ is meant to serve as ballast material to better approximate conditions of an actual wash in a commercial washer. Next, the three fabric swatches were each immediately immersed in 10 ml of DEB and vortexed for 10 s to extract any remaining bacteria. The DEB solution was then dilution plated to calculate the percent reduction in viability by the sanitizer. To recover any bacteria not extracted from the swatches, the swatches were incubated in 10 ml of DEB for 5 h at 37 °C and 150 RPM.

### Carrier count control

*S. aureus* in 30 μl DEB was inoculated onto outer shell swatches, dried for 30 min at 37 °C, and washed by high-speed vortexing (10 s or 10 min) in 50 ml test tubes containing 10 ml of sanitizer diluted 1:6803 in water for maximal contact with the sanitizer to determine the number of viable organisms remaining in the swatch after vortex washing (referred to as carrier count controls in ASTM E2274). Replicate swatches were washed with water only for comparison. After vortex washing, all swatches were rinsed by pipetting 50 ml of sterile water on both sides of the swatch. Each swatch was then placed in a sterile Erlenmeyer flask containing 10 ml of DEB and incubated for 5 h at 37 °C and 150 RPM to extract any viable bacteria that had remained on the swatch after vortexing. The number of viable bacteria that were extracted from a swatch was calculated based on the doubling time (45 min) of *S. aureus* in DEB and the number of CFUs obtained after dilution plating on Dey Engley Agar (DEA). The log_10_ reduction in viability of *S. aureus* on swatches washed with sanitizer was calculated as the difference between the number of viable *S. aureus* extracted from swatches washed with water and the number of viable *S. aureus* extracted from swatches washed with the sanitizer. The assay was repeated four times using Gemini™, two times with Advance™, and performed once using Pioneer™ or cotton fabric at two contact times. Swatches were initially sterilized by autoclaving, inoculated and processed under sterile conditions in a laminar flow hood, and handled with sterilized instruments.

### In house washer/extractor tests and laundering procedure

Four Gemini™ or four Pioneer™ swatches inoculated with 2.9 × 10^7^ *S. aureus* (stored 24 h /4 °C before use to simulate shipping time as described in the shipping survival test) were pinned inside the underarm areas (left and right) of two turnout gear coats (inner lining removed as describe in NFPA 1851). To serve as cross-contamination indicators, four sterile Gemini™ or four sterile Pioneer™ swatches were aseptically pinned adjacent to the inoculated swatches. The wash loads were adjusted to include a total of six pieces of zipped-up turnout gear (20.34 lb./9.22 kg load) and placed into a 40 lb./18.14 kg capacity Milnor washer/extractor (Model 30015V7J, Pellerin Milnor Corporation, Kenner, LA), and washed with 0.8 oz. of concentrated sanitizer and 8 gal absorbed water/9.5 gal free water to achieve ~ 70% of the load capacity, allowing the clothing to move freely. A 10 min soak/sanitizing cycle at 105 °F was programed into the washer/extractor followed by a 4-min spin cycle extraction at low speed (G-force < 100). After the washing was complete, the swatches were air-dried and stored in a sterile and sealed petri dish at 4 °C for 24 h to simulate the return shipping. Each swatch was subsequently placed in a sterile Erlenmeyer flask containing 10 ml of DEB for 5 h at 37 °C and shaken at 150 RPM. The number of viable cells in each swatch was then calculated as described in the Carrier count control section above.

### Survival of *S. aureus* on outer shell swatches during shipping

A shipping simulation test of *S. aureus* inoculated swatches was designed to determine whether *S. aureus* will remain viable over 7 days, the estimated time to ship, field test in a washer/extractor loaded with turnout ballast material, and return the swatches for analysis. Thirty-two Gemini™ swatches inoculated with 2.9 × 10^7^ *S. aureus* were divided into eight groups of four swatches, each group of swatches to be processed immediately (Day 0) or in 24 h intervals (Days 1–7). The test was done in duplicate. An uninoculated swatch was included in each group to assess for cross-contamination during a wash. Each group of five swatches (four inoculated + one uninoculated) was stored in a sterile, parafilm-sealed petri dish at 4 °C to simulate transport time ranging from zero to seven days. At 24 h intervals, the five swatches from a group were each placed in a sterile Erlenmeyer containing 10 mL DEB for five hours at 37 °C and shaken at 150 RPM. The number of viable cells in each swatch was then calculated as described in the Carrier count control section above.

### Data analysis

All analyses were performed using JMP version 13 (SAS Institute, Cary NC). Analysis of the effects of time and swatch type following washing were performed using two-way analysis of variance (ANOVA). Survival of bacteria over time was analyzed using a one-way ANOVA, and comparisons of DNA content recovered from cells were performed using a t-test. Pearson correlation coefficients were calculated to assess the correlation between qPCR and the viability assessments. All differences were considered significant at *p* < 0.05. Percent reduction and log_10_ reduction were calculated as described below:

$$ \%\mathrm{Reduction}=\frac{\left(\mathrm{CFU}\ \mathrm{Extracted}\ \mathrm{from}\ \mathrm{Water}\ \mathrm{Washed}\ \mathrm{Swatch}\right)-\left(\mathrm{CFU}\ \mathrm{Extracted}\ \mathrm{from}\ \mathrm{Sanitizer}\ \mathrm{Washed}\ \mathrm{Swatch}\right)}{\left(\mathrm{CFU}\ \mathrm{Extracted}\ \mathrm{from}\ \mathrm{Water}\ \mathrm{Washed}\ \mathrm{Swatch}\right)}\times 100 $$

$$ {\mathrm{Log}}_{10}\mathrm{Reduction}={\log}_{10}\left(\mathrm{Average}\ \mathrm{CFU}\ \mathrm{Water}\ \mathrm{Washed}\ \mathrm{Swatches}\right)-\left(\mathrm{Average}\ \mathrm{CFU}\ \mathrm{Sanitizer}\ \mathrm{Washed}\ \mathrm{Swatches}\right) $$

## Results

### Bacterial contamination and effect of sterilization on outer shell fabrics

**Fig. 1:**
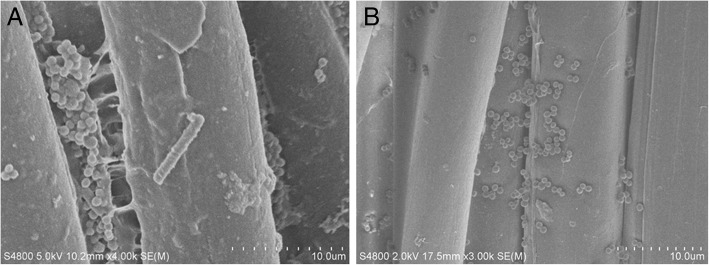
SEM of bacterial contaminants still present on decommissioned turnout gear jackets. Random sections of the **a** Gemini™ and **b** Advance™ outer shells were aseptically cut from a decommissioned turnout gear jacket and processed for SEM. The representative SEM shows the presence of cocci and rod structures embedded in matrices of exopolysaccharide, a critical step in biofilm formation

**Fig. 2:**
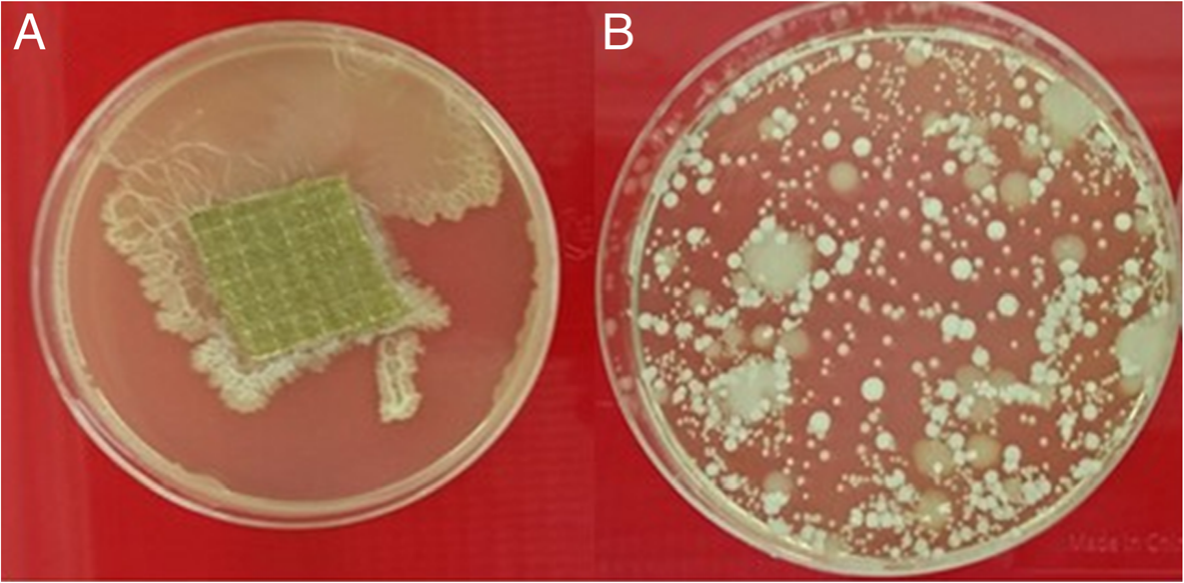
Viable bacterial cells present on laundered Gemini™ fabric. A new roll of Gemini™ obtained from the manufacturer was laundered in-house in a commercial washer/extractor five times prior to its use in subsequent experiments. A random swatch of this fabric was incubated at 37 °C for 48 h on TSA. **a** Shows the presence of viable bacteria remaining on the swatch. **b** Serial dilution plating of cells recovered from swatch show a variety of bacterial types

**Fig. 3:**
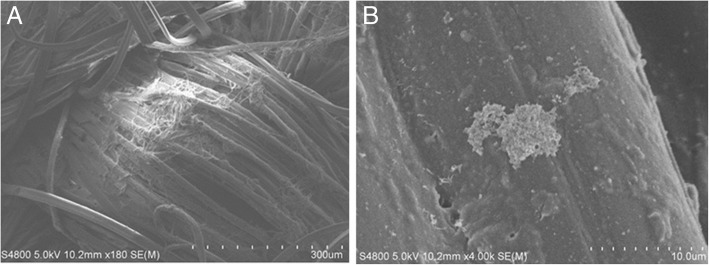
SEM examination of Gemini™ after scouring or autoclaving to disinfect the fabric. Gemini™ fabric was scoured according to ASTM E2274–16 or autoclaved. **a** Microscopic texture of the scoured outer shell fabric displaying entangled smaller filaments due to denaturation of the fabric. **b** Inactivated microbial cells still attached to the fiber after autoclaving. Each tic mark in the magnification scale represents 1/10th of the total μm length indicated

**Fig. 4:**
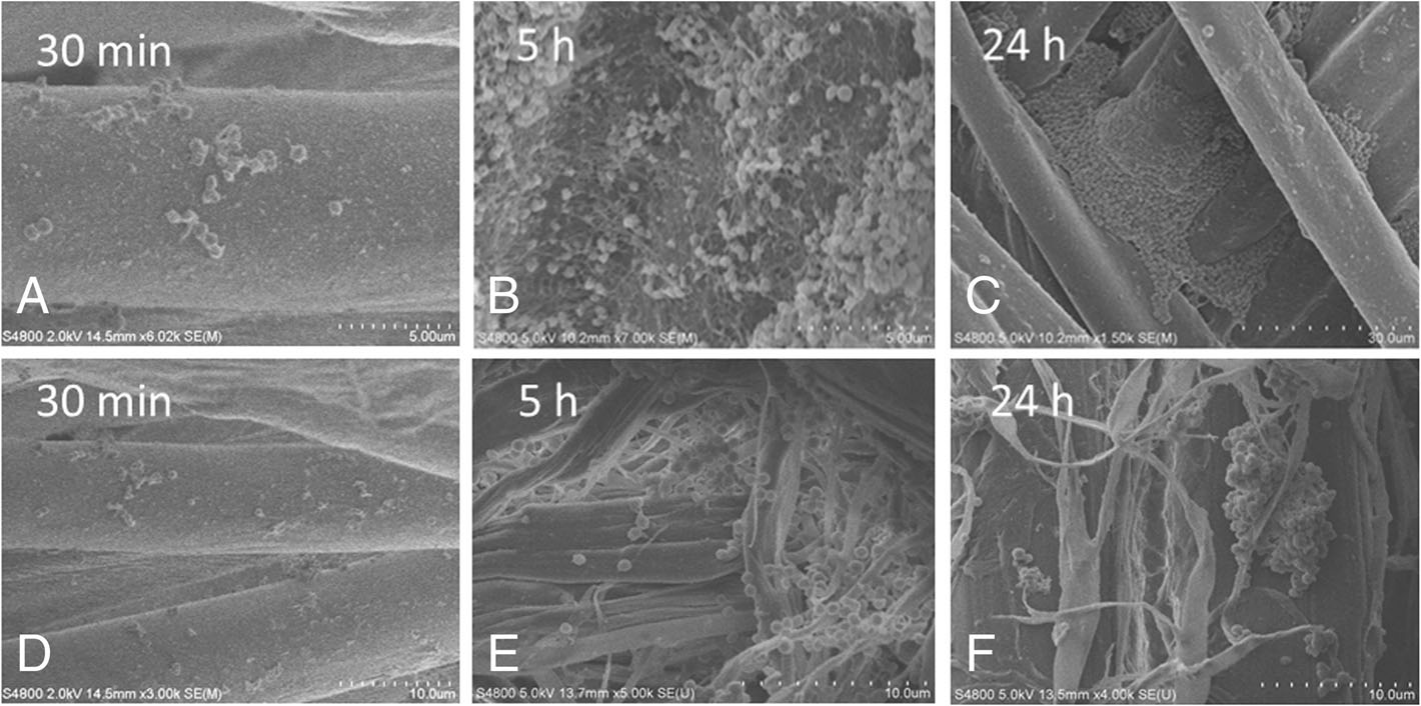
SEM images showing the three stages of biofilm growth on Gemini™. (30 min) Adhesion and accumulation of *S. aureus* on Gemini™ after inoculation. (5 h) Consolidation of the interface by formation of polymer bridges. (24 h) Colonization of Gemini™ and the encapsulation of *S. aureus* cells in an exopolysachharide matrix. Replicate images at two different magnifications are shown. Each tic mark in the magnification scale represents 1/10th of the total μm length indicated

**Fig. 5:**
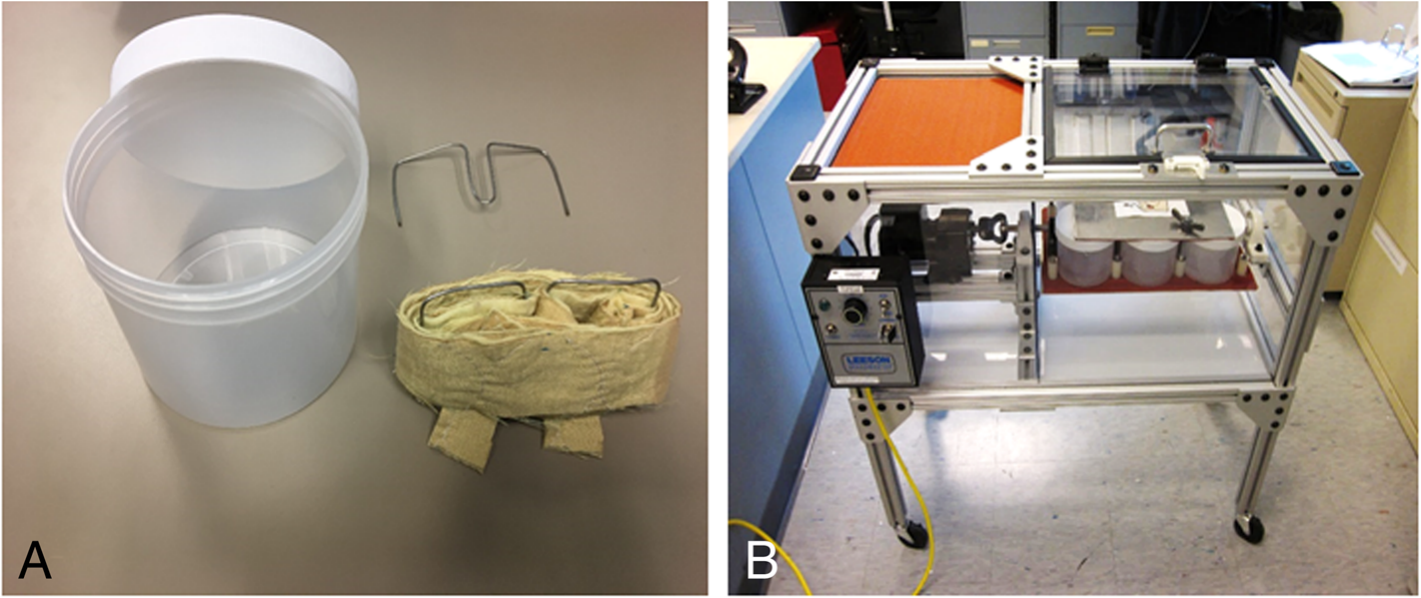
ASTM E2274 compliant laundry tumbler. A laundry tumbler that conforms to ASTM E2274–09 and simulates the washing of fabric swatches in top-loading automatic washing machines. The tumbler has six 1 L jars that are removable for sterilization. **a** The swatches are inserted (two swatches shown) into turnout gear fabric that is wrapped around a small metal spindle which is then placed in the jar with sanitizer. **b** The jars are placed inside the laundry tumbler and rotated at 60 rpm for the prescribed time

SEM examination prior to fabric sterilization revealed cocci present in biofilms and rod-like bacteria attached to the fibers of decommissioned turnout gear jackets with Gemini™ (Fig. 1a) and Advance™ (Fig. 1b) outershells. Similarly, fabric swatches from a bolt of Gemini™ fabric incubated on TSA plates yielded up to 10^2^ CFU/swatch (Fig. 2). Structural changes to the Gemini™ fabric in the form of masses of entangled smaller fiber conglomerations were seen after scouring to kill existing bacteria according to ASTM E2274–16 (Fig. 3a). Autoclaving did not affect the fabric structure but clumps of presumed dead microbial cells were evident (Fig. 3b). Gemini™ was inoculated with 1.9 × 10^7^ *S. aureus* in 30 μl DEB to assess for potential biofilm formation when worn by fire fighters responding to emergencies that would expose them to this bacteria. After inoculation of an aliquot of *S. aureus* onto a swatch, the cells were allowed to dry for 30 min at room temperature (Fig. 4, 30 min). Placing the inoculated swatch into DEB media for 5 h shows the formation of polymer bridges between the cells and the swatch (Fig. 4, 5 h) and after 24 h (Fig. 4, 24 h), colonization and biofilm formation occurs. These results show that fire fighter turnout gear has the potential to support *S. aureus* biofilm formation in as little as 24 h if the outer shell fabric is exposed to environmental conditions rich in these microbes.

### Effectiveness of outer shell sanitization using ASTM E2274 protocol

Our initial test of the sanitizer’s bacteriocidal efficacy showed that directly spiking *S. aureus* into sanitizer resulted in complete loss of viability of *S. aureus* in as little as 10 s as no viable cells were recovered following pelleting of the cells by centrifugation (Table 1). We then tested bacteriocidal efficacy using the ASTM E2274 protocol which was established to determine the effectiveness of sanitizers, laundry detergents, and other additives for use in top-loading automatic washer/extractors. To assess the efficacy of this protocol for use in evaluating the sanitizer’s ability to disinfect fire fighter turnout gear, we constructed a laundry tumbler (Fig. 5) according to the design specified in ASTM E2274 and washed swatches inoculated with *S. aureus***.** Three experiments were performed with Gemini™ swatches inoculated with 1.3 × 10^7^, 5.9 × 10^6^ or 5.4 × 10^7^ CFU/swatch (Table 2). The recoveries of viable bacteria from swatches washed with sterile water (control) were 2.3 × 10^6^ (16%), 2.4 × 10^6^ (41%) and 2.0 × 10^6^ (4%), respectively. Washing with sanitizer reduced the number of viable cells in the wash to zero. Rinsing the water-washed swatches with sterile water recovered an additional 2.1 × 10^6^ (16%), 4.2 × 10^6^ (71%) and 3.1 × 10^6^ (6%), respectively, of viable bacteria, but none from the swatches washed with the sanitizer.

**Table 1:** Bacteriocidal efficacy of the sanitizer

Duration of vortexing in water or sanitizer	Inoculum (CFU)	Recovery of viable cells exposed to water (CFU)/^a^SD	^a^Recovery of viable cells exposed to sanitizer (CFU)	Percentage reduction of viable cells (%)	Log reduction of viable cells
10 Seconds	2.1 × 10^7^	1.4 × 10^7^/1.3 × 10^6^	0	100	6.8
1 Minute		1.6 × 10^7^/1.4 × 10^6^	0	100	6.8
10 Minutes		1.6 × 10^7^/1.2 × 10^6^	0	100	6.2

Experiment repeated three times with three replicate swatches for each vortex time

^a^*SD* standard deviation

**Table 2:** Effectiveness of Gemini™ sanitization using ASTM E2274 protocol

Exp. no.	^a^Inoculum (CFU/Swatch)	Recovery of viable cells in the wash water (control) (CFU)	Recovery of viable cells in sanitizer wash (CFU)	Recovery of viable cells after rinsing the water washed swatch (control) (CFU / ^b^SD)	Recovery of viable cells after rinsing the sanitizer washed swatch (CFU)
Exp 1	1.3 × 10^7^	2.3 × 10^6^	0	2.1 × 10^6^/5.1 × 10^5^	0.83
Exp 2	5.9 × 10^6^	2.4 × 10^6^	0	4.2 × 10^6^/2.7 × 10^5^	0
Exp 3	5.4 × 10^7^	2.0 × 10^6^	0	3.1 × 10^6^/5.1 × 10^5^	0.17

^a^Three replicate swatches per experiment

^b^*SD* standard deviation

### Development of a methodology to assess cleaning practices by ISPs

The above experiments showed that the ASTM E2274 protocol does not provide a clear picture of how many viable cells remain on the outer shell fabric after washing with sanitizer. To better address this, Gemini™, Advance™, Pioneer™, and cotton swatches were inoculated with *S. aureus* (carrier count controls in ASTM E2274), placed in conical test tubes containing water or sanitizer, and washed by high-speed vortexing for 10 s or 10 min. This was done to assess how well the fabrics could be disinfected under ideal wash conditions. Neither water nor sanitizer was very effective at physically removing *S. aureus* from outer shell fabrics and removal was highly variable. After 10 s of vortex-washing of Gemini™ or Advance™ with water, only 2 and 3%, respectively, of the viable cells inoculated on these fabrics were recovered in the wash water (Table 3). Increasing the vortex-washing to 10 min only increased the physical removal of *S. aureus* from Gemini™ to 12% and Advance™ to 21% (Table 4). Removal of *S. aureus* from uncoated Pioneer™ and cotton after only 10 s of vortex-washing was far more efficient (29 and 42%, respectively). Similarly, after washing the fabrics with sanitizer for 10 s or 10 min, qPCR showed that most of the *S. aureus* remained on the Gemini™ and Advance™ swatches although the amount that was physically washed off was highly variable (Table 3).

**Table 3:** Efficiency of removing *S. aureus* from outer shell fabrics

Duration of vortexing in water or sanitizer	Swatch type	% viable cells recovered in water washed swatch /^a^SD	% viable cells recovered in sanitizer washed swatch	% PCR products recovered in water washed swatch /^a^SD	% PCR products recovered in sanitizer washed swatch /^a^SD
10 Seconds	Gemini™	1.5 /1.2	0	13.6 /13.0	6.5 /4.2
	Advance™	3.0 /1.2	0	0.9 /0.9	10.4 /13.9
	Pioneer™	29 /6.3	0	8.3 /6.6	18 /27
	Cotton	41.7 /14.8	0	1.4 /^b^ND	6.6 /7.2
10 Minutes	Gemini™	11.7 /7.3	0	1.7 /1.7	0.5 /0.8
	Advance™	20.4 /21.3	0	1 /1.2	1.9 /1.6
	Pioneer™	11 /1.8	0	10.2 /4.8	4.3 /3.4
	Cotton	85.6 /15.6	0	3.9 /5.0	0.8 /0.4

Gemini™ experiments repeated 4 times with 3 replicate swatches each

Advance™ experiments repeated 2 times with 3 replicate swatches each

Pioneer™ experiment performed 1 time with 3 replicate swatches

Cotton experiment performed 1 time with 3 replicate swatches

^a^*SD* standard deviation

^b^*ND* not determined as two of the three replicate swatches were below detection

**Table 4:** Reduction in viability of *S. aureus* still present on outer shell fabrics after washing with sanitizer

Duration of vortexing in water or sanitizer	Swatch type	Exp. no.	Inoculum (CFU/ Swatch)	Recovery of viable cells from water washed swatch (CFU /^a^SD)	Recovery of viable cells from sanitizer washed swatch (CFU /^a^SD)	Percent reduction
10 Seconds (Carrier Count Control)	Gemini™	1	1.7 × 10^7^	2.5 × 10^5^ /1.5 × 10^5^	1.4 × 10^5^ /1.8 × 10^5^	46
	Gemini™	2	1.9 × 10^7^	8.1 × 10^5^ /3.8 × 10^5^	1.8 × 10^5^ /1.6 × 10^5^	77
	Gemini™	3	3.7 × 10^7^	5.1 × 10^4^ /1.2 × 10^4^	6.6 × 10^3^ /1.4 × 10^3^	87
	Gemini™	4	3.4 × 10^7^	1.9 × 10^5^ /1.2 × 10^5^	3.5 × 10^4^ /1.7 × 10^4^	82
	Advance™	1	2.5 × 10^7^	4.3 × 10^4^ /1.2 × 10^4^	1.7 × 10^2^ /1.1 × 10^2^	99.6
	Advance™	2	2.5 × 10^7^	4.3 × 10^4^ /6.5 × 10^3^	4.7 × 10^2^ /9.7 × 10^1^	98.9
	Pioneer™	1	2.4 × 10^7^	4.0 × 10^5^ /4.8 × 10^4^	1.1 × 10^1^ /1.3 × 10^1^	100
	Cotton	1	2.4 × 10^7^	1.5 × 10^6^ /5.8 × 10^4^	2.3 × 10^2^ /3.2 × 10^2^	99.9
10 Minutes (Carrier Count Control)	Gemini™	1	3.3 × 10^7^	3.2 × 10^5^ /2.6 × 10^5^	1.2 × 10^5^ /7.3 × 10^4^	63
	Gemini™	2	1.4 × 10^7^	9.0 × 10^5^ /3.1 × 10^4^	9.1 × 10^3^ /4.3 × 10^3^	99
	Gemini™	3	3.5 × 10^7^	1.6 × 10^6^ /7.9 × 10^5^	7.9 × 10^5^ /8.4 × 10^5^	51
	Gemini™	4	3.0 × 10^7^	4.8 × 10^6^ /9.2 × 10^5^	1.9 × 10^6^ /1.6 × 10^6^	61
	Advance™	1	2.1 × 10^7^	4.3 × 10^4^ /1.4 × 10^5^	1.9 × 10^2^ /7.7 × 10^2^	99.5
	Advance™	2	2.5 × 10^7^	4.3 × 10^4^ /1.7 × 10^4^	2.1 × 10^3^ /5.8 × 10^2^	95
	Pioneer™	1	3.9 × 10^7^	8.2 × 10^4^ /3.2 × 10^4^	0	100
	Cotton	1	2.2 × 10^7^	1.2 × 10^6^ /4.8 × 10^5^	0	100
Washer/Extractor	Swatch type	Exp. no.	Inoculum (CFU/ Swatch)	Recovery of viable cells from unwashed swatch (CFU /^a^SD)	Recovery of viable cells from sanitizer washed swatch (CFU /^a^SD)	Percent reduction
Test 1	Gemini™	1	2.9 × 10^7^	1.6 × 10^6^ /5.7 × 10^5^	4.2 × 10^3^ /1.4 × 10^3^	99.7
Test 2	Pioneer™	1	2.9 × 10^7^	1.3 × 10^6^ /5.8 × 10^5^	0	100

Carrier count swatches were vortex-washed for 10 s or 10 min, and then incubated in DEB for 5 h to elute remaining cells

All carrier count experiments are average of 3 replicate swatches per experiment

Washer/extractor test of *S. aureus* inoculated Gemini swatches are average of 4 swatches pinned to two turnout gear jackets

Washer/extractor test of *S. aureus* inoculated Pioneer swatches are average of 3 swatches pinned to two turnout gear jackets

^a^*SD* standard deviation

**Fig. 6:**
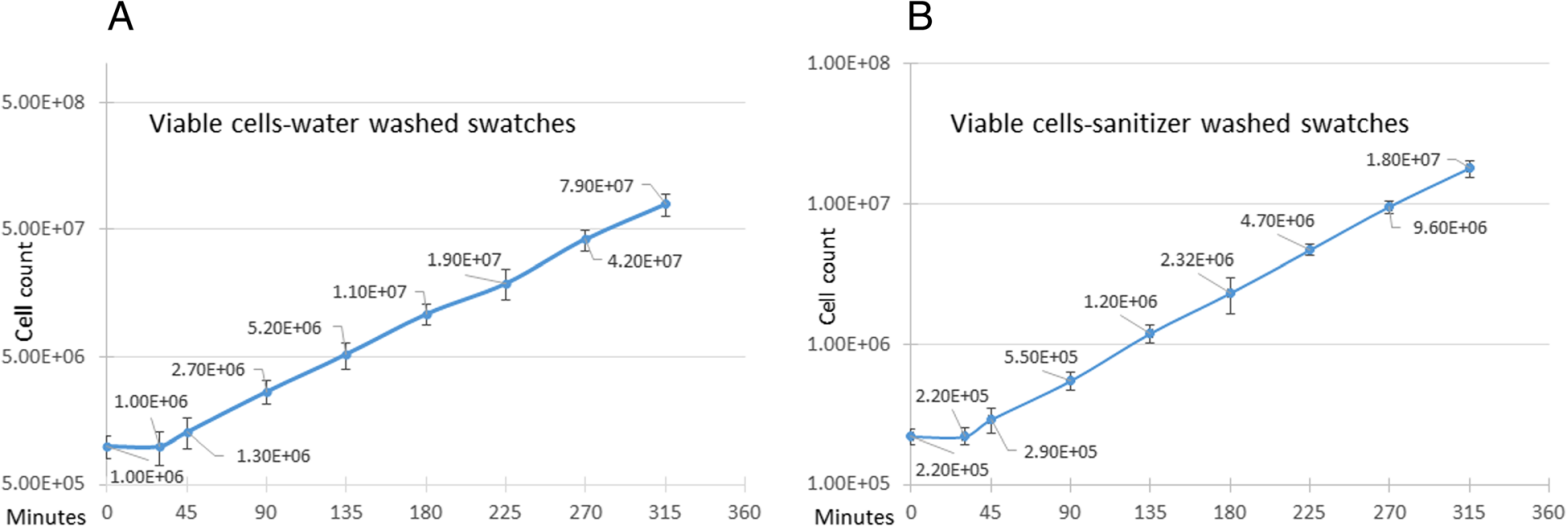
Recovery and growth of viable *S. aureus* during 5 h incubation in DEB. Gemini™ swatches were inoculated with 2.5 × 10^7^ *S. aureus,* washed with either **a**  water or **b** sanitizer for 10 min, and transferred to DEB for 5 h. Aliquots of the cultures were plated on TSA at 0, 30, 45, 90, 135, 180, 225, 270, and 315 min after transfer to DEB. The number of viable cells recovered from the swatches is shown. The experiment was repeated three times, three replicate swatches per experiment, and standard deviation is shown

To determine whether viable bacteria remained on the swatches washed with sanitizer, the swatches were then incubated in DEB media to elute any remaining *S. aureus.* Since any viable *S. aureus* that is eluted will continue to divide over the course of culturing in DEB, the number of bacteria that had been extracted was calculated by taking into account a lag time of 30–45 min before resumption of log growth with a doubling time of 45 min in DEB. This was initially confirmed by inoculating swatches and exposing them to either water or sanitizer for 10 min, transferring the swatches to DEB, and plating aliquots of the growth media onto TSA plates at 45 min intervals to determine the number of viable cells appearing in the growth media (Fig. 6). These results showed that ~ 4% of the cells that were inoculated onto Gemini™ swatches was initially eluted after washing with water for 10 min. The assumption we then had to make was that ~ 4% of the cells inoculated onto the sanitizer washed swatches would also initially be eluted. Moreover, viable cells released from sanitizer washed swatches could conceivably experience a longer lag time before resuming logarithmic growth, thus, resulting in an underestimate of the number of viable cells that were resistant to the sanitizer. However, as shown, the lag time of viable cells exposed to sanitizer was approximately the same (30–45 min) as that of water exposed cells before resuming their 45 min doubling time (Fig. 6). We, therefore, concluded that any viable cells remaining on the swatches following exposure to sanitizer were not damaged as to extend their lag time nor affect their doubling time, and during the 5 h incubation in DEB, both water exposed and sanitizer exposed cells would have undergone approximately six doublings.

**Fig. 7:**
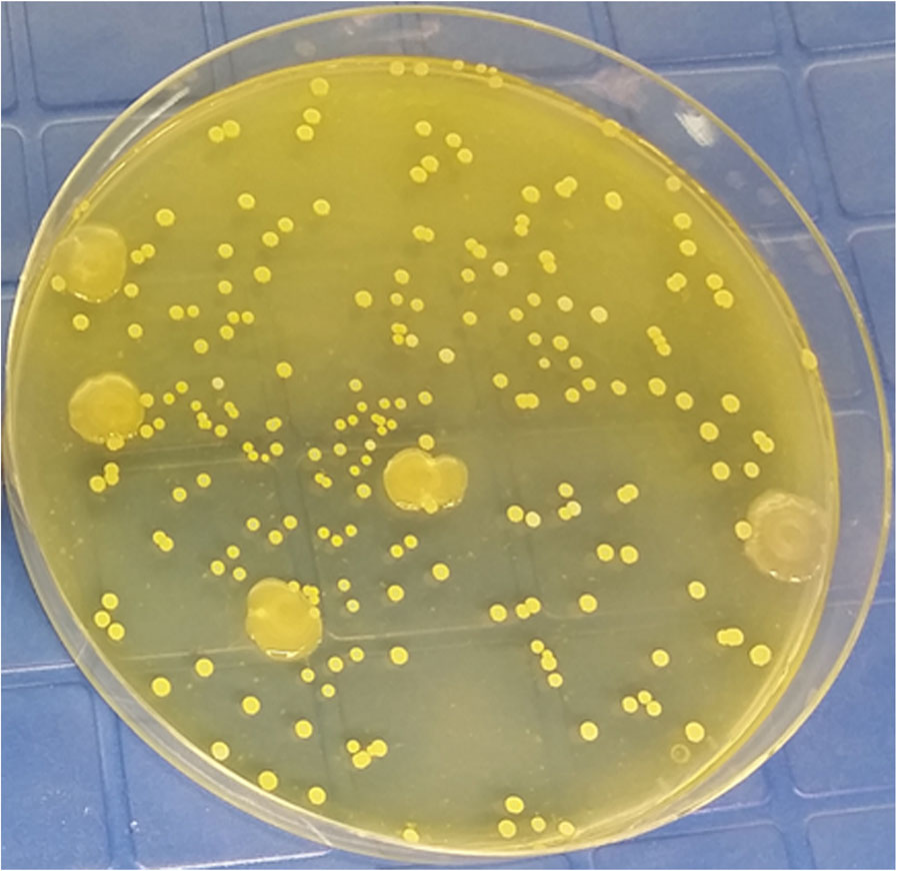
Sanitizer-resistant *P. aeruginosa.* Example of *P. aeruginosa* (large, circular, raised and undulate colonies) recovered as a cross contaminant from a decommissioned turnout gear jacket that was laundered along with a Gemini™ swatch inoculated with *S.aureus* (small, round and golden-yellow colonies)

The guidelines in NFPA1851 describe general cleaning procedures for fire fighter turnout gear, however, there are no procedures or requirements to demonstrate whether such cleaning practices actually result in the removal and/or disinfection of microbial contaminants. To address whether an ISP that cleans turnout gear actually has in place a procedure for efficient disinfection, we modified the ASTM E2274 protocol by including carrier count control swatches in an actual wash of turnout gear in a commercial washer. Since we showed (Fig. 6) that the majority of *S. aureus* remains on the turnout gear swatches, each swatch was then placed in a sterile Erlenmeyer flask containing 10 ml of DEB and incubated for 5 h at 37 °C and 150 RPM to extract any viable bacteria that were not killed by the sanitizer. After vortex-washing with sanitizer for 10 s, on average, 73% (Recovery of Viable cells from Sanitizer Washed Swatch divided by Recovery of Viable Cells from Water Washed Swatch) of the *S. aureus* that remained on the Gemini™ swatch was killed (Table 4). Increasing the vortex-washing to 10 min did not improve the efficiency of the sanitizer as 69% of the *S. aureus* that remained on the Gemini™ was killed. After 10 s of vortex-washing Advance™, 91% of the *S. aureus* that remained on the swatch was killed and in the 10 min-vortex washing, 97% of the bacteria was killed. Scoured Pioneer™ was the most efficiently sanitized (as efficient as cotton fabric) as essentially 100% of the remaining *S. aureus* on the swatch was killed. Complete sterility was obtained for both Pioneer™ and cotton after 10 min of vortex-washing as no bacterial growth was observed from either swatch type after as much as 48 h of incubation in DEB broth (results not shown).

To then determine the efficiency of sanitization that could be obtained in a standard wash of turnout gear in a commercial washer, Gemini™ swatches inoculated with *S. aureus* were pinned to two decommissioned turnout jackets and washed along with four other jackets which served as ballast. Analysis of the washed swatches showed that the efficiency of sanitization was 99.7% (Recovery of Viable cells from Sanitizer Washed Swatch divided by Recovery of Viable Cells from Unwashed Swatch) (Table 4). Similarly, Pioneer™ swatches were completely sanitized (100% killing). Since all *S. aureus* that is washed off the swatches was killed by the sanitizer (Table 1), then all swatches washed in the commercial washer were essentially 100% sanitized.

QuantitativePCR analysis of the cells that remained on the swatch after washing with sanitizer was performed and the results are shown in Table 5. Although both dead and viable cells are expected to be eluted from a swatch, growth for 5 h prior to PCR analysis would amplify the number of viable cells (~ 6 doublings) and PCR analysis of this culture would reflect mostly *femB* copies from them. On average, 88% (Number of *femB* Copies from Viable Cells Remaining on Sanitizer Washed Swatch divided by Number of *femB* Copies from Viable Cells Remaining on Unwashed Swatch) of the *S. aureus* cells that remained on the Gemini™ swatches after vortex-washing with sanitizer for 10 s were killed. Again, increasing the vortex-washing to 10 min did not improve efficiency of the sanitizer (80% killing). Approximately 98% of the *S. aureus* remaining on the Advance™ swatches was killed after only 10 s of vortex-washing, and killing reached 99.5% after 10 min of washing. Uncoated Pioneer™ and cotton were the most efficiently sanitized as essentially complete sterility was obtained after only 10 s of vortex-washing. Analysis of eluted cells from Gemini™ and Pioneer™ swatches washed in the commercial washer/extractor showed 99.6 and 99.5% killing, respectively. These results show that the percentage of viable cells remaining on the swatches as assessed by plating on TSA (Table 4) correlates well with the number of *femB* copies obtained from these viable cells (Table 5).

**Table 5:** Reduction in *FemB* copies from viable *S. aureus* still present on outer shell fabrics after washing with sanitizer

Duration of vortexing in water or sanitizer	Swatch type	Exp. no.	Inoculum (*femB* copies/ swatch)	*femB* copies from viable cells remaining on water washed swatch (*femB* /^a^SD)	*femB* copies from viable cells remaining on sanitizer washed swatch (*femB* /^a^SD)	Percent reduction
10 Seconds (Carrier Count Control)	Gemini™	1	2.2 × 10^6^	1.0 × 10^5^ /1.5 × 10^5^	3.1 × 10^4^ /8.0 × 10^4^	69
	Gemini™	2	2.0 × 10^6^	3.6 × 10^4^ /1.5 × 10^4^	5.7 × 10^3^ /4.7 × 10^3^	84
	Gemini™	3	2.0 × 10^7^	5.6 × 10^3^ /3.5 × 10^3^	2.0 × 10^1^ /4.4 × 10^1^	99.6
	Gemini™	4	6.0 × 10^6^	5.4 × 10^4^ /4.6 × 10^4^	1.2 × 10^3^ / 8.9 × 10^2^	98
	Advance™	1	1.0 × 10^7^	4.8 × 10^4^ /5.0 × 10^4^	4.5 × 10^1^ /2.2 × 10^1^	100
	Advance™	2	1.0 × 10^7^	8.2 × 10^4^ /4.7 × 10^4^	3.7 × 10^3^ /5.2 × 10^3^	95
	Pioneer™	1	2.2 × 10^5^	2.8 × 10^4^ /2.3 × 10^4^	2.9 × 10^1^ /4.7 × 10^1^	99.9
	Cotton	1	1.1 × 10^5^	2.3 × 10^4^ /2.3 × 10^4^	1.6 × 10^2^ /3.0 × 10^2^	99.3
10 Minutes (Carrier Count Control)	Gemini™	1	1.1 × 10^7^	6.7 × 10^4^ /5.0 × 10^4^	8.6 × 10^3^ /9.2 × 10^3^	87
	Gemini™	2	2.3 × 10^8^	7.2 × 10^5^ /4.6 × 10^5^	1.7 × 10^5^ /2.7 × 10^5^	77
	Gemini™	3	4.0 × 10^7^	8.1 × 10^4^ /2.0 × 10^4^	1.6 × 10^4^ /1.8 × 10^4^	80
	Gemini™	4	1.5 × 10^8^	9.7 × 10^4^ /2.0 × 10^4^	2.5 × 10^4^ /3.8 × 10^4^	74
	Advance™	1	1.2 × 10^7^	6.4 × 10^4^ /2.5 × 10^5^	4.7 /9.7	100
	Advance™	2	5.5 × 10^6^	7.6 × 10^4^ /4.3 × 10^5^	9.3 × 10^2^ /1.2 × 10^4^	99
	Pioneer™	1	1.1 × 10^7^	2.5 × 10^5^ /1.1 × 10^5^	1.0 × 10^3^ /3.5 × 10^2^	99.6
	Cotton	1	9.1 × 10^6^	2.1 × 10^6^ /4.0 × 10^5^	1.9 × 10^2^ /1.7 × 10^2^	100
Washer/extractor	Swatch type	Exp. no.	Inoculum (*femB* copies/Swatch)	*femB* copies from viable cells remaining on unwashed swatch (*femB* /^a^SD	f*emB* copies from viable cells remaining on fabric sanitizer washed swatch (*femB* /^a^SD)	Percent reduction
Test 1	Gemini™	1	9.6 × 10^7^	1.6 × 10^6^ /1.7 × 10^6^	6.8 × 10^3^ /2.0 × 10^4^	99.6
Test 2	Pioneer™	2	8.7 × 10^7^	4.7 × 10^5^ /2.1 × 10^6^	3.1 × 10^2^ / 2.0 × 10^4^	99.9

Carrier count swatches were vortex-washed for 10 s or 10 min, and then incubated in DEB for 5 h to elute remaining cells

All carrier count experiments are average of 3 replicate swatches per experiment

Washer/extractor test of *S. aureus* inoculated Gemini swatches are average of 4 swatches pinned to two turnout gear jackets

Washer/extractor test of *S. aureus* inoculated Pioneer swatches are average of 3 swatches pinned to two turnout gear jackets

^a^*SD* standard deviation

**Fig. 8:**
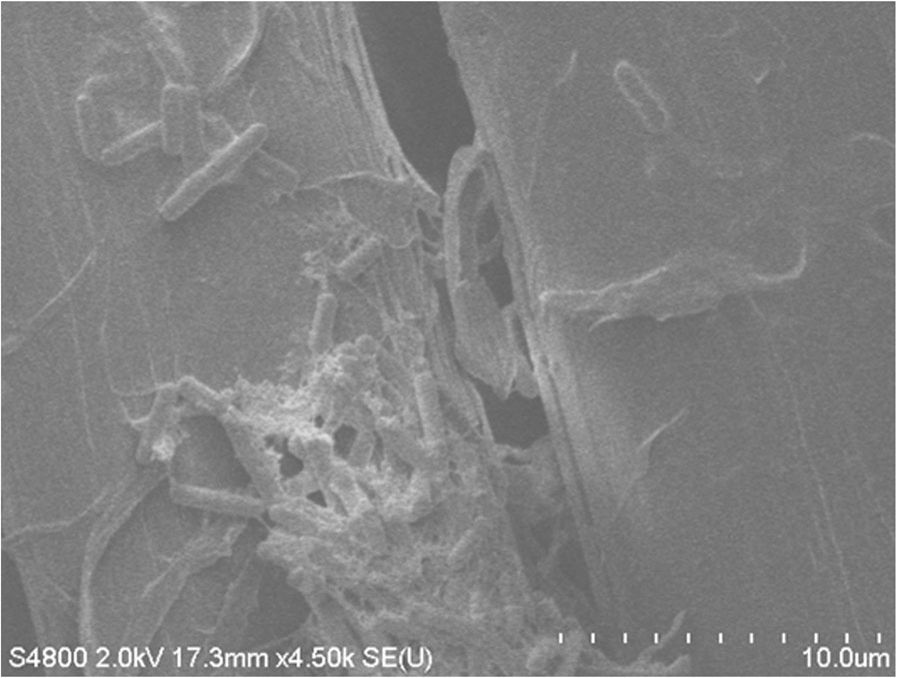
SEM of Sanitizer-resistant *P. aeruginosa* growing on Gemini™. The strain was inoculated onto a Gemini™ swatch that was subsequently incubated in DEB for 5 h. SEM shows the rod-shaped *P. aeruginosa* encased in exopolysaccharide (the “glycocalyx”) which facilitated the adherence and colonization of Gemini™ fabric fibers and subsequent biofilm production

A DDAC-resistant strain of *P. aeruginosa* was recovered from the sterile swatches that were placed adjacent to the *S. aureus*-inoculated swatches pinned to the machine-washed fire fighter turnout jackets. The recovered strain was inoculated into 2 ml of diluted sanitizer, allowed to stand for 1–10 min, and then dilution plated to confirm that no loss in viability was evident (data not shown). *P. aeruginosa* was identified based on its rod-shape under SEM, its large colony morphology (Fig. 7) growth characteristics in DEB (*P. aeruginosa* does not ferment dextrose and forms a pellicle on the surface of the broth, and confirmed by qPCR identification of the *regA* chromosomal gene. *P. aeruginosa* was found on Gemini™ and Pioneer™ at approximately 10^2^ CFU/swatch. Gemini™ was inoculated with this *P. aeruginosa* strain and after 5 h incubation in DEB media, biofilm formation was evident (Fig. 8).

### Shipping survival test

**Fig. 9:**
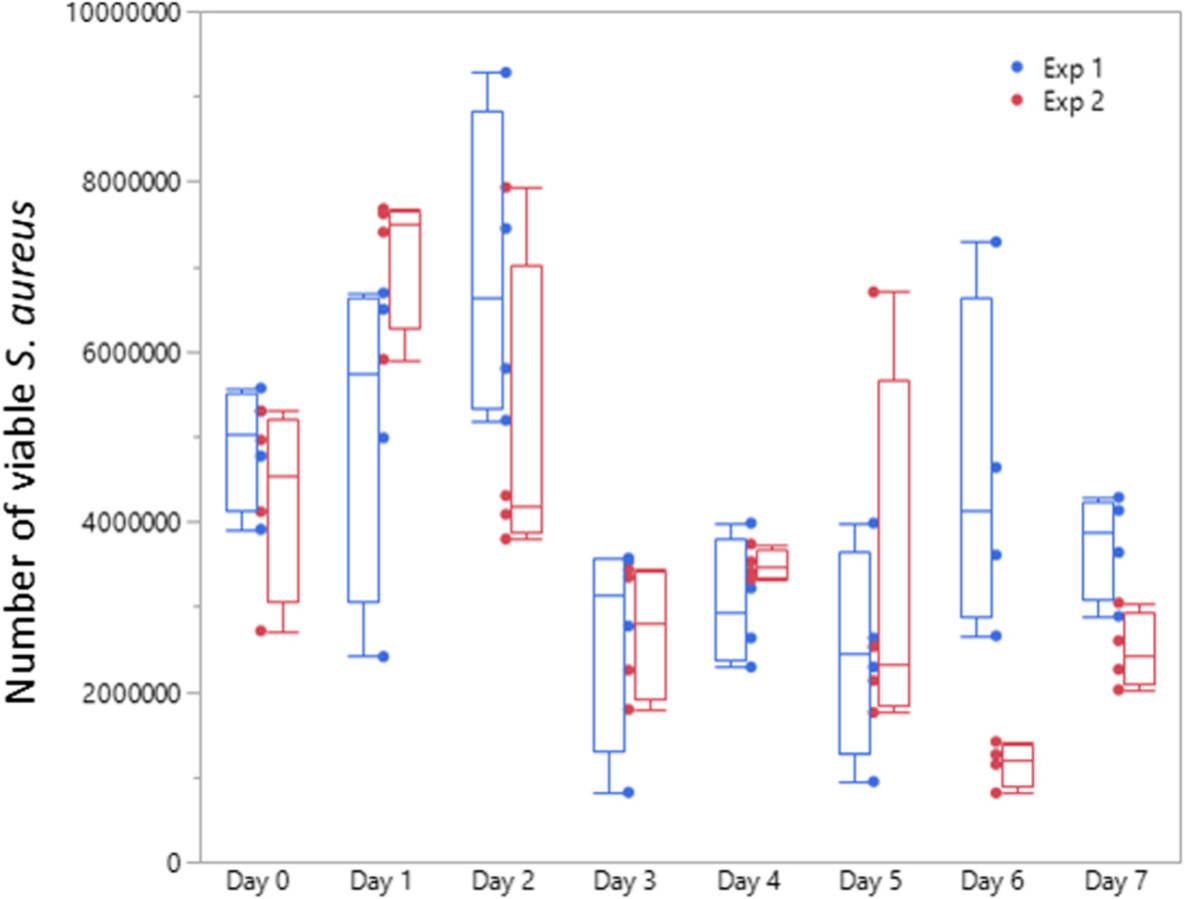
Survival of *S. aureus* on swatches during shipping. Gemini™ swatches were inoculated with 2.9 × 10^7^ *S. aureus* and either processed immediately (Day 0) or stored for 1–7 days at 4 °C before processing for the number of viable *S. aureus* that remained on the swatches. For each time point, 4 inoculated and 1 uninoculated Gemini™ swatches were processed. Two independent experiments were performed. Sampling variation was noted between some of the days. Whisker Box Plot shows the median (middle line in each bar) the 25th and 75th percentiles (the limits of the box), and the upper max and min points along with each data point for each day and individual experiment

To validate a commercial wash facility’s ability to efficiently wash fire fighter turnout gear, the ISP would include *S. aureus* contaminated swatches of outer shell fabric in their standard wash and return the washed swatches to a test facility that would assess whether the swatches were sanitized. During shipping to and from the ISP, the viability of *S. aureus* would need to be consistently maintained in order for the assay to be valid. Therefore, we performed a mock shipping experiment to ensure that inoculated swatches could be stored for several days before use. Gemini™ swatches inoculated with *S. aureus* were stored for 0–7 days at 4 °C and placed in DEB for 5 h to assess the number of viable bacteria still present. Over the course of 0–7 days, an average of 4.2 × 10^6^ CFU (SD = 1.9 × 10^6^) viable cells were recovered from the swatches in one test experiment and 3.7 × 10^6^ CFU (SD = 2.1 × 10^6^) in a second test (Fig. 9). These results show that swatches contaminated with *S. aureus* can be stored up to 7 days (the time to ship/test/return swatches) and can be effectively used to assess a vendor’s wash procedure.

## Discussion

One aim of this study was to evaluate the effectiveness of an EPA registered sanitizer to disinfect the outer shell of fire fighter turnout that may be contaminated with *S. aureus* and reduce the risk of transfer of this bacteria to the fire fighter. The sanitizer contains a quaternary ammonium compound, DDAC, at 50% by weight. It was previously reported that DDAC binds tightly through ionic and hydrophobic interactions to the membrane of *S. aureus* and inserts its hydrophobic tail into the cell’s lipid bilayer that leads to leakage of intracellular constituents including genomic DNA [17].

ASTM E2274 is based on the study by Petrocci and Clarke [18] that proposed a method to test the antimicrobial effectiveness of laundry additives and is most amenable to disinfection of fabrics such as cotton and linen that are porous and readily absorb water and laundry additives. Although the sanitizer™ performed well on Cotton and Pioneer™ in our study (> 3 log reduction of viable *S. aureus*), it cannot be generalized as a universal fabric sanitizer. While cotton’s hollow hydrophilic structure and its ability to draw a sanitizer along the fibers through capillary action aids the sanitizers’ biocide action, the hydrophobic solid fibers of Gemini™ and Advance™ resist water absorption and, likely, hinders access of the sanitizer to any microbial masses developed on the fibers. ASTM E2274 also calls for a “sterile exposure chamber” which serves as a “model top-loading washer/extractor” and focuses on the direct antimicrobial action of the test solution on the targeted bacterial cells but disregards the effect of existing bacterial cells within a washer/extractor. Discovery of *P. aeruginosa* strains adapted to persist on inanimate surfaces and that survive against high concentrations of quaternary ammonium have been documented since the 1970s [19]. In our study, a DDAC-resistant strain of *P. aeruginosa* was presumably released from the decommissioned turnout jackets that we included as ballast in the washer/extractor tests and led to cross-contamination of the sterile Gemini™ swatches pinned to that turnout gear.

Moreover, our analysis of decommissioned turnout gear jackets showed the presence of bacterial biofilm that persisted after laundering. In general for most if not all bacteria, biofilm formation can be divided into three stages: 1) attachment of planktonic bacterial cells to the surface, 2) colonization and maturation of the biofilm, and 3) subsequent detachment and dispersion of cells to new sites. [20, 21]. The specific mechanisms for biofilm formation often differ among bacterial species. *S. aureus* attaches to abiotic surfaces mostly through hydrophobic or electrostatic interactions and can involve bacterial proteins including autolysin or teichoic acids, whereas, attachment to biotic surfaces is facilitated through host matrix proteins referred to as MSCRAMMs (microbial surface components recognizing adhesive matrix molecules) [22]. *P. aeruginosa* attachment can involve bacterial molecules including adhesins, flagella, type IV pili, Psl polysaccharide, and lipopolysaccharides [23, 24]. Biofilm colonization and maturation in *S. aureus* involves production of the extracellular polymer matrix which contains host factors, polysaccharides, proteins and extracellular DNA (eDNA) [25]. Similarly, *P. aeruginosa* also incorporates polysaccharides (alginate, Pe, and PsL) to stabilize the biofilm and DNA which appears to play a structural role [26]. Tolerance to antimicrobials in the mature biofilm is the result of delayed diffusion of the antimicrobial deep into the biofilm and other mechanisms that involve upregulation of specific genes such as the *pmr*-LPS-modification system and the MexAB-OprM efflux pump genes [24]. Resistance to sanitizers may, thus, involve processes that delay their diffusion into the biofilm or other mechanisms. We also observed staphylococcal biofilm under SEM of Gemini™ that was incubated in DEB for 24 h demonstrating the ability of *S. aureus* to adsorb, consolidate and colonize on this fabric. The initial adhesion of *S. aureus* cells on Gemini™ begins very early, within 30 min of inoculation, and is likely facilitated by the close proximities of the fabrics fibers. Moreover, SEM analysis of Gemini™ suggests that biofilm has begun to form after 5 h incubation in DEB, demonstrating the potential for rapid biofilm formation in the field when worn by fire fighters.

Our study also proposes a new full-scale laundry machine test method, designed especially to determine the efficacy of detergents and sanitizers in the fabric cleaning process for individual washer/extractors. To validate the ability to efficiently wash fire fighter turnout gear, the ISP will include *S. aureus* contaminated outer shell fabric swatches in their standard wash and return the washed swatches to a test facility that will assess if the swatches were sanitized. During shipping to and from the vendor, the viability of *S. aureus* would need to be consistently maintained in order for the assay to be valid, and as such, this was confirmed in our study. This method was developed to be a portable, simple, inexpensive, and reliable tool in assessing the effectiveness of a cleaning process while accommodating variables encountered in the field such as the specific interaction between the microorganism strains and the fabric surface, wash cycle time and temperature, contaminated matrices (feces, blood, sweat or vomit), and pH or wash water hardness. It is also conceivable that each individual washer/extractor may have a unique microbial biofilm formation which may result in the contamination of textiles during laundering at low temperature [27], and further investigations of turnout gear cleaning facilities are recommended. Although this new test method is only representative for a particular washer/extractor among several washers at a commercial facility, our proposed field method depicts a more realistic washer/extractor test that is simple to implement in all the facility’s washers and can be readily adapted to include testing for a wide variety of bacteria species.

Fire fighter exposure to contaminated personal protective equipment (PPE) is an increasing concern for long-term fire fighter health. Cancer and other diseases resulting from chronic exposures to persistent harmful contamination found on their PPE have become the leading industry issue. While general cleaning procedures have been established in the National Fire Protection Association (NFPA) 1851, *Standard on Selection, Care, and Maintenance of Protective Ensembles for Structural Fire Fighting and Proximity Fire Fighting*, there are no requirements that demonstrate whether current cleaning practices will adequately remove contaminants from fire fighter PPE. Many manufacturer gear cleaning recommendations are vague and most cleaning product/process claims are unsubstantiated regarding contaminant removal effectiveness. This study was funded by the Fire Protection Research Foundation (FPRF), and partners on this grant included three Fire Service Organizations, six Fire Departments, and three ISPs for washing fire fighter gear. The overall goal was to reduce fire fighter exposure to harmful contaminants on PPE by establishing standardized methods that could be used to determine the decontamination effectiveness of cleaning methods. Changes to the NFPA 1851 by providing guidelines for effectively removing both chemical and biological contaminants from PPE are expected to be the end result. In this study, the outer shell of turnout gear was evaluated as a priority since this water/chemical resistant barrier is the most critical element of this PPE. A logical extension of this proposal led to a second funded FPRF grant to develop methods for assessing cleaning that can be applied to items such as helmets, hoods, gloves, footwear, SCBA as part of firefighter PPE, but which may also be extended to seat covers or tools. These items remain significant threats for firefighter long-term contact with contaminants but yet involve different materials, mechanisms of contamination, and approaches for cleaning that differ from garment outer shells. The findings gained from subsequent work will fill gaps in relevant standards with additional requirements and will further result in new best practices that lead to improved cleanliness and hygiene within the fire services for contamination control.

## Conclusion

Although the sanitzer was not 100% effective at disinfecting *S. aureus* on all outer shell fabric types washed by vortex-washing, it was 100% effective when used in the commercial washer/extractor in our facility. This sanitizer, however, was not effective at preventing cross-contamination of outer shell fabrics with a DDAC-resistant strain of *P.aeruginosa* and, therefore, such strains will continue to pose a problem. Our study demonstrated that the efficacy of a sanitizer depends on the type of outer shell fabric to be washed and that the potential presence of biofilm formation within an ISP’s washer/extractors may also affect the cleanliness of the washed turnout gear. Our improved methodology for assessing the effectiveness of cleaning outer shell fabrics is an easy, fast, and reliable alternative for assessing the effectiveness of cleaning fire fighter turnout gear by ISPs and may help reduce the occupational exposure to fire fighters from microbial contaminants.

## Abbreviations

AATCC: American Association of Textile Colourists and Chemists
ASTM: American Society for Testing Materials
CA-MRSA: Community-acquired MRSA
CDC: Centers for Disease Control and Prevention
DDAC: Didecyl dimethyl ammonium chloride
DEA: Dey Engley Agar
DEB: Dey Engley Broth
EPA: Environmental Protection Agency
FPRF: Fire Protection Research Foundation
HA-MRSA: Hospital-associated MRSA
MRSA: Methicillin-resistant *S. aureus*
MSSA: Methicillin sensitive *S. aureus*
NA: Nutrient agar
NFPA: National Fire Protection Association
NIOSH: National Institute for Occupational Safety and Health
qPCR: Quantitative polymerase chain reaction
SEM: Scanning electron microscopy
TSA: Tryptic soy agar
TSB: Tryptic soy broth
